# Integrated Analyses Identify *CDH2* as a Hub Gene Associated with Cisplatin Resistance and Prognosis in Ovarian Cancer

**DOI:** 10.3390/ijms27020713

**Published:** 2026-01-10

**Authors:** Jun-Yi Xu, Mao-Qi Tian, Rui Yang, Zi-Xuan Li, Zi-Heng Lin, Yu-Fei Wang, Yu-Hang Chu, Wei-Ning Sun, Ya-Mei Wang

**Affiliations:** 1 School of Basic Medical Sciences, Capital Medical University, No. 10 Xitoutiao, You An Men, Beijing 100069, China; jeffxuccmu@mail.ccmu.edu.cn (J.-Y.X.); tmq374025166@163.com (M.-Q.T.); ruiyang@mail.ccmu.edu.cn (R.Y.); l3422189483@163.com (Z.-X.L.); linziheng@mail.ccmu.edu.cn (Z.-H.L.); wangyufei@mail.ccmu.edu.cn (Y.-F.W.); 18640102849@163.com (Y.-H.C.); weiningsun@mail.ccmu.edu.cn (W.-N.S.); 2 Department of Biochemistry and Molecular Biology, School of Basic Medical Sciences, Capital Medical University, No. 10 Xitoutiao, You An Men, Beijing 100069, China

**Keywords:** ovarian cancer, cisplatin resistance, cadherin 2 (CDH2), gene set enrichment analysis, immune infiltration, drug screening

## Abstract

Ovarian cancer (OC), the third most common gynecologic malignancy, is characterized by high mortality largely driven by chemotherapy resistance, leading to recurrence and metastasis. Using transcriptomic data from GSE73935, we constructed a weighted gene co-expression network and identified eight hub genes (*IGF1R*, *CDH2*, *PDGFRA*, *CDKN1A*, *SHC1*, *SPP1*, *CAV1* and *FGF18*) associated with cisplatin resistance, among which *CDH2* emerged as the most clinically relevant candidate. *CDH2* demonstrated moderate diagnostic potential (AUC = 0.792) and was markedly upregulated in cisplatin-resistant A2780/CP70 cells. Independent validation using clinical single-cell RNA-seq data (GSE211956) confirmed its selective enrichment in resistant tumor cell subpopulations. Gene set enrichment analysis linked elevated *CDH2* expression to p53 signaling, DNA replication, nucleotide excision repair, and Toll-like receptor pathways, with qPCR supporting upregulation of key downstream genes in resistant cells. Immune deconvolution further indicated that high *CDH2* expression correlated with increased infiltration of NK cells, Tregs, macrophages, and neutrophils, and immunohistochemistry verified CDH2 overexpression in cisplatin-resistant tissues. In addition, virtual screening and drug sensitivity profiling identified several FDA-approved agents with potential relevance to *CDH2*-associated drug response. These findings indicate that *CDH2* may serve as a candidate marker associated with cisplatin response in OC, and its association with immune cell infiltration provides further insight into mechanisms potentially underlying chemoresistance.

## 1. Introduction

Ovarian cancer (OC) is one of the most common and lethal gynecologic malignancies worldwide. According to global cancer statistics in 2022, approximately 324,398 new cases and 206,839 deaths were reported [1,2]. Owing to the lack of early diagnostic markers, nearly 70% of patients are diagnosed at advanced stages, resulting in a 5-year survival rate of less than 50% [3]. Standard treatment for OC consists of cytoreductive surgery followed by platinum-based chemotherapy, among which cisplatin remains a cornerstone agent. Cisplatin exerts antitumor activity primarily by forming DNA adducts that disrupt DNA replication and transcription, ultimately inducing tumor cell death [4]. However, despite favorable initial responses, most patients eventually develop acquired cisplatin resistance, which represents a major cause of disease recurrence and poor prognosis. Cisplatin resistance in OC is a complex and multifactorial process involving altered drug transport, enhanced DNA damage repair, dysregulated apoptosis, and remodeling of the tumor microenvironment. Increasing evidence also implicates epithelial–mesenchymal transition (EMT), cancer stemness, immune evasion, and metabolic reprogramming as key contributors to cisplatin-resistant phenotypes [5,6,7,8]. Although targeted therapies such as PARP inhibitors and anti-angiogenic agents have improved outcomes in selected patient subsets, reliable biomarkers that predict or monitor cisplatin resistance remain lacking, limiting the implementation of precision treatment strategies.

High-throughput transcriptomic profiling has facilitated the identification of molecular alterations associated with cisplatin resistance. Previous studies have reported that dysregulation of DNA repair pathways and oncogenic signaling cascades, including the PI3K–AKT–mTOR pathway, is frequently enriched in cisplatin-resistant OC [9,10,11,12]. However, traditional differential expression analyses primarily focus on individual genes and may fail to capture coordinated gene networks underlying drug resistance. Weighted gene co-expression network analysis (WGCNA) is a systems biology approach that identifies biologically relevant gene modules based on co-expression topology, enabling the discovery of key regulatory genes associated with complex phenotypes such as cisplatin resistance [13]. While WGCNA has been widely applied in cancer research [14,15,16,17], its use in elucidating gene networks specifically associated with cisplatin resistance in OC remains limited.

The rapid development of single-cell RNA sequencing (scRNA-seq) has further advanced the study of tumor heterogeneity and drug resistance by enabling transcriptomic profiling at single-cell resolution. Unlike bulk RNA sequencing, scRNA-seq allows the identification of resistant tumor subpopulations and cell type-specific transcriptional programs associated with cisplatin resistance, providing critical insights into tumor evolution and therapeutic failure [18,19,20,21,22]. Integrating bulk transcriptomic analysis with single-cell validation therefore represents a powerful strategy to dissect the molecular basis of cisplatin resistance.

In this study, we analyzed the GSE73935 dataset, which comprises transcriptomic profiles of A2780 ovarian cancer cells with experimentally induced resistance to cisplatin. Using WGCNA, we constructed gene co-expression networks and identified key modules and hub genes associated with the cisplatin-resistant phenotype. To enhance clinical relevance, we further validated candidate genes using an independent clinical scRNA-seq dataset (GSE211956), enabling evaluation of their expression patterns at single-cell resolution across tumor cell populations. Through this integrated analytical framework, we aimed to identify robust biomarkers associated with cisplatin resistance in OC and to provide mechanistic insights into resistance-related molecular and cellular processes.

## 2. Results

### 2.1. Differentially Expressed Genes Identification

**Figure 1:**
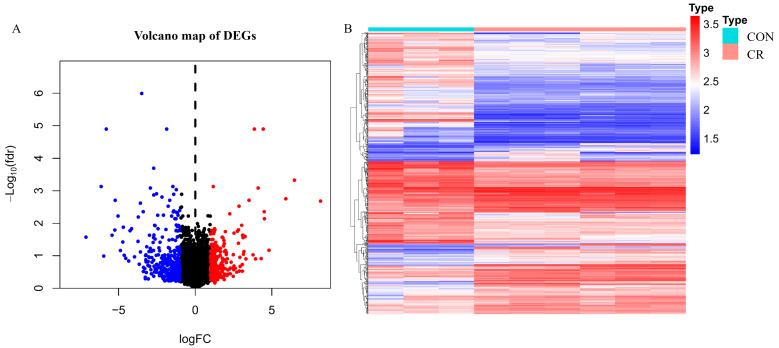
Identification of DEGs associated with cisplatin resistance. (**A**) Volcano plot of the GSE73935 dataset showing differentially expressed genes between cisplatin-resistant (CR) and control (CON) A2780 ovarian cancer cells. Each dot represents an individual gene; red dots indicate significantly upregulated genes and blue dots indicate significantly downregulated genes in the CR group based on the defined cut-off criteria. (**B**) Heatmap illustrating the expression patterns of DEGs between cisplatin-resistant (CR) and control (CON) groups. Genes are hierarchically clustered based on expression similarity. Increased and decreased expression levels are shown in light red and light blue, respectively.

Differences in gene expression between cisplatin-treated and untreated groups in samples after screening for GSE73935 [23] (n = 48) (clinical information detailed in Supplementary Table S1A) were analyzed using R language. The data set was filtered with false discovery rate (FDR) < 0.05, |logFC| > 1 with *p*-value below 0.05. 623 differentially expressed genes (DEGs) were identified between the cisplatin resistant (CR) groups and control groups (CON), with 372 transcripts showing reduced expression and 251 demonstrating elevated expression levels, as detailed in Supplementary Table S1B. Differential expression analysis was additionally assessed between each group and the control group. The paclitaxel resistant (PX) group has 845 differentially expressed genes, the topotecan resistant (TO) group has 19, and the doxorubicin resistant (DO) group has 337, with detailed information provided in the Supplementary Table S1B. Volcano plots and heat maps were drawn using the ggplot2 package in R (Figure 1A, B), where red indicates upregulated genes and blue indicates downregulated genes.

### 2.2. WGCNA Construction and Hub Module Identification

**Figure 2:**
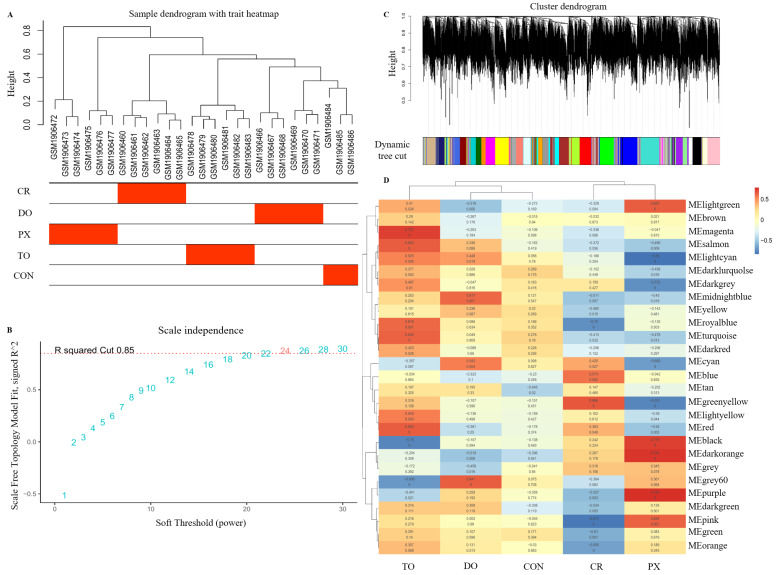
WGCNA of the GSE73935 dataset. (**A**) Hierarchical clustering dendrogram of the 27 samples based on gene expression profiles, with corresponding treatment traits shown below. Samples include cisplatin-resistant (CR, n = 6), doxorubicin-resistant (DO, n = 6), paclitaxel-resistant (PX, n = 6), topotecan-resistant (TO, n = 6), and untreated control groups (CON, n = 3). (**B**) Determination of the soft-thresholding power (β) for network construction. Scale-free topology fit index and mean connectivity were evaluated, and β = 24 was selected to achieve approximate scale-free topology. (**C**) Gene clustering dendrogram based on topological overlap dissimilarity (1 − TOM), with genes grouped into distinct co-expression modules. (**D**) Heatmap of module–trait relationships showing Pearson correlations between module eigengenes and resistance phenotypes for different chemotherapeutic agents (cisplatin, doxorubicin, paclitaxel, and topotecan). Color intensity indicates the strength and direction of correlations, with corresponding correlation coefficients and *p*-values shown in each cell.

WGCNA facilitates the identification of disease-associated gene modules based on coordinated expression patterns, thereby enhancing the detection of key regulatory genes. Clustering analysis on the expression matrix of 27 samples yielded five experimental groups, supporting the study design and confirming sample consistency (Figure 2A). We selected a soft-thresholding power of β = 24 to ensure a scale-free network topology, achieving an R^2^ value greater than 0.8 (Figure 2B). Using the WGCNA algorithm, the gene set was subsequently divided into 27 modules, each represented by a distinct color (Figure 2C). Module-phenotype associations were analyzed via heatmap visualization (Figure 2D), identifying co-expression modules with statistically significant correlations to clinical phenotypes. Notably, the green-yellow (r = 0.666, *p* < 0.01, n = 70) and blue (r = 0.574, *p* < 0.01, n = 140) module showed strong positive correlations with cisplatin resistance. In contrast, the pink (r = −0.811, *p* < 0.01, n = 83) and royal blue modules (r = −0.72, *p* < 0.01, n = 42) exhibited significant negative correlations. Notably, the identified modules exhibited weaker or non-significant correlations with resistance to other chemotherapeutic agents, further supporting their relative specificity to cisplatin resistance rather than a generalized multidrug-resistant phenotype. All correlation *p*-values were under 0.05, as detailed in Supplementary Table S1C, and the gene names of each key module are detailed in Supplementary Table S1D.

### 2.3. Protein–Protein Interaction Network Analysis of DEGs

**Figure 3:**
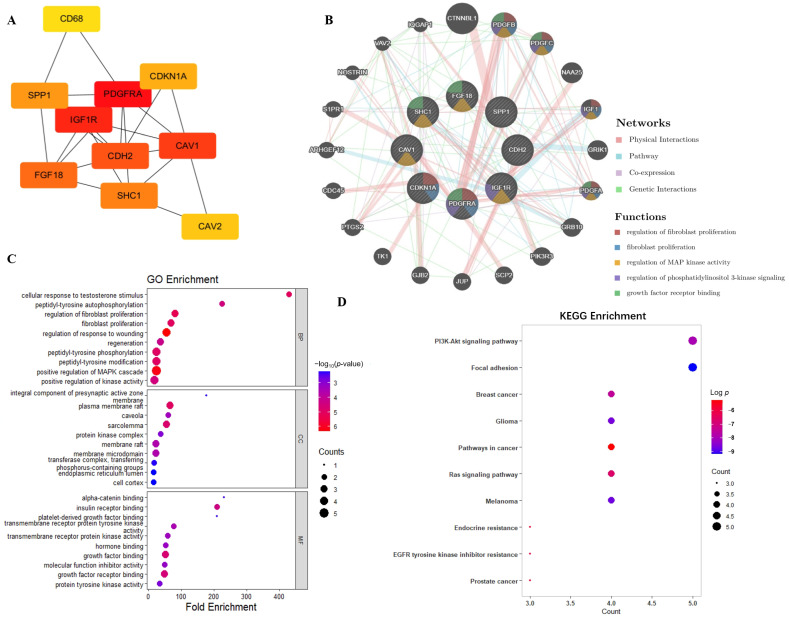
PPI network and hub genes’ functional enrichment analyses. (**A**) PPI network constructed using the STRING database, illustrating the interaction landscape among hub genes identified from cisplatin resistance–related WGCNA modules. Nodes represent proteins, and edges indicate predicted or experimentally validated interactions. (**B**) Functional annotation of hub genes derived from the most cisplatin-relevant WGCNA modules. (**C**) GO enrichment analysis of hub genes, including biological process, molecular function, and cellular component categories. The color gradient from blue to red represents the −log_10_ (*p*-value), where red indicates a higher value and greater statistical significance. (**D**) KEGG pathway enrichment analysis of hub genes, highlighting pathways potentially involved in cisplatin resistance. The color gradient represents the log *p* values, with red indicating higher statistical significance.

The genes present within each related module were then subjected to an intersection with the DEGs. Venn analysis identified intersecting genes across four co-expression modules: Blue (24 genes), Green-yellow (8 genes), Pink (22 genes), and Royal blue (12 genes), aggregating to 66 candidate genes (Supplementary Table S1E). These genes were input into the STRING website to generate a protein–protein interaction (PPI) network diagram, which was then displayed in Cytoscape (Version: 3.10.0). Notably, 8 significant protein-coding genes are centrally located within the network: *IGF1R*, *CDH2*, *PDGFRA*, *CDKN1A*, *SHC1*, *SPP1*, *CAV1*, and *FGF18* (Figure 3A). *IGF1R*, *CDH2*, and *PDGFRA* belong to the green-yellow module, and *CDKN1A*, *SHC1* belong to the blue module, which are strongly positively correlated with cisplatin resistance. In contrast, the pink module, which includes *CAV1*, *FGF18*, and the royal blue module, which contains *SPP1*, are negatively correlated with cisplatin resistance. (the maximal clique centrality (MCC) score of each gene is shown in Supplementary Table S1F). Functional enrichment analysis was further conducted via the GeneMANIA platform [24] revealing a multifaceted interactome among candidate genes. The PPI network demonstrated three principal association types: physical interactions (41.24%), co-expression patterns (21.86%), and pathway co-regulation (36.87%) (Figure 3B). Kyoto Encyclopedia of Genes and Genomes (KEGG) and Gene Ontology (GO) analyses of these genes revealed their association with several path- ways (Figure 3C,D), including the PI3K-protein kinase B (Akt) signaling pathway, and breast cancer, the Ras signaling pathway, focal adhesion, as well as their relevance to biological processes related to learning functions. Furthermore, these genes are involved in the positive regulation of kinase activity, peptidyl- tyrosine phosphorylation and autophosphorylation, mitogen-activated protein kinase (MAPK) cascade, along with functions related to protein kinase complexes and transmembrane receptor protein tyrosine kinase activity. These pathways and molecular functions play critical roles in cell signaling, proliferation, and survival. Notably, aberrant activation of these signaling cascades is often associated with tumor drug resistance.

### 2.4. mRNA Expression Levels and Kaplan–Meier Survival Analysis of Candidate Hub Genes

**Figure 4:**
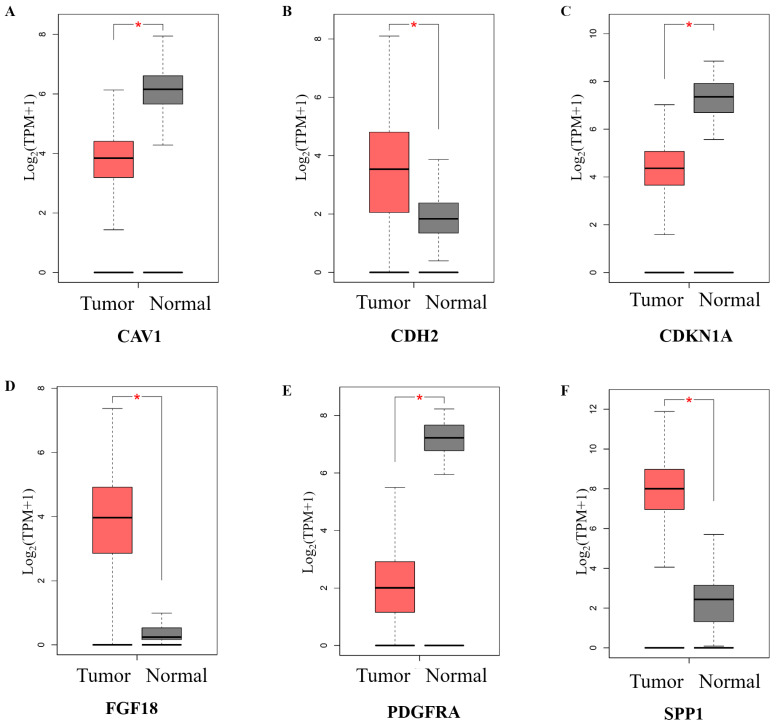
Differential mRNA expression of hub genes in ovarian cancer and normal ovarian tissues. mRNA expression levels of six hub genes were compared between ovarian cancer (OC; n = 426) and normal ovarian tissues (n = 88) using data from the GEPIA database. Panels (**A**–**F**) correspond to the individual hub genes. The box plots represent the expression levels with Tumor and Normal shown on the X-axis. The asterisk (*) indicates |log_2_ fold change| >1 and *p* < 0.01. Statistical significance was assessed by the GEPIA built-in One-way ANOVA analysis, and *p* < 0.01 was considered significant.

**Figure 5:**
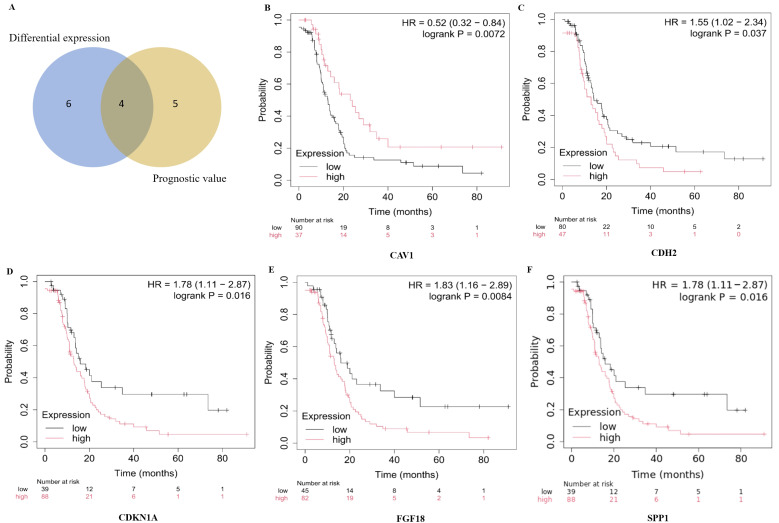
Prognostic significance of candidate hub genes in OC. (**A**) Venn diagram illustrating the overlap between genes exhibiting significant differential expression and those associated with progression-free survival (PFS), identifying four overlapping hub genes. (**B**–**F**) Kaplan–Meier survival curves showing the PFS of patients stratified by high and low expression levels of the five prognostically significant hub genes, as assessed using the Kaplan–Meier Plotter database.

Comparative study of mRNA expression levels for eight candidate hub genes between 426 OC samples and 88 normal ovarian tissue samples were performed via the GEPIA database (http://gepia.cancer-pku.cn/ (accessed on 23 July 2024)). Among these, six genes exhibited significant differential expression (*p* < 0.01) (Figure 4A–F). Specifically, *CDH2*, *FGF18*, and *SPP1* were significantly upregulated, whereas *CAV1*, *CDKN1A*, and *PDGFRA* were downregulated in OC tissues. The prognostic relevance of these six differentially expressed hub genes was further evaluated using Kaplan–Meier survival analysis. Five genes were significantly associated with progression-free survival (PFS). To identify robust candidates with both expression alteration and prognostic significance, an integrative cross-filtering strategy was applied (Figure 5A), resulting in four overlapping hub genes: *CDH2*, *FGF18*, *CAV1*, and *SPP1*. Kaplan–Meier (K-M) survival curves for progression-free survival (PFS) are shown in Figure 5B–F. Notably, patients with elevated expression of *CDH2*, *FGF18*, *SPP1*, and reduced expression of *CAV1*, exhibited significantly shorter PFS (*p* < 0.05).

### 2.5. Identification and Validation of the Hub Gene CDH2

**Figure 6:**
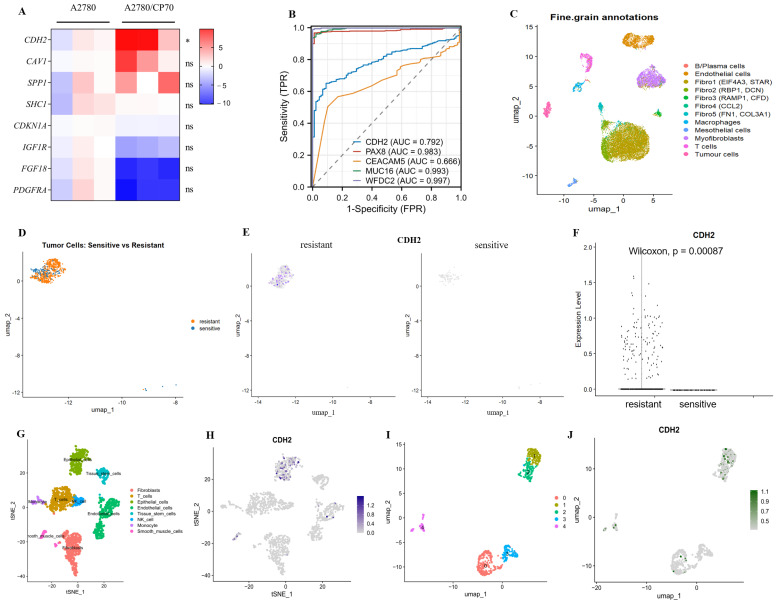
Identification and validation of *CDH2* as a hub gene in OC. (**A**) RT–qPCR analysis of *CDH2* expression in cisplatin-sensitive A2780 cells and cisplatin-resistant A2780/CP70 cells, with GAPDH used as the internal control. Data are presented as mean ± SD from three independent experiments; statistical significance was assessed using a paired *t*-test. The asterisk (*) indicates a statistically significant difference (* *p* < 0.05). (**B**) ROC curves assessing the diagnostic performance of *CDH2*, *PAX8*, *CEACAM5*, *MUC16*, and *WFDC2* expression in ovarian cancer. (**C**–**J**) scRNA-seq analysis of based on the GSE211956 dataset. (**C**) UMAP visualization of all single cells from ovarian cancer patients, annotated by cell type based on canonical markers expression and Single R; different colors represent distinct cell types. (**D**) UMAP visualization of tumor cells extracted from cisplatin-sensitive and cisplatin-resistant groups. (**E**) Feature plot showing *CDH2* expression patterns in tumor cells from sensitive and resistant groups; the color gradient represents the gene expression level (grey indicates low expression, while purple indicates high expression). (**F**) Dot plot of *CDH2* expression levels between the two groups (adjusted *p* = 0.0147, Wilcoxon *p* = 0.00087). Each black dot represents an individual cell. The horizontal lines indicate the median expression level for each group. (**G**) UMAP visualization of all single cells from cisplatin-resistant patient samples, annotated by cell type. (**H**) Feature plot of *CDH2* expression in resistant patient samples; the color gradient represents the gene expression level (grey indicates low expression, while purple indicates high expression). (**I**) UMAP visualization of epithelial, smooth muscle, and endothelial cells from cisplatin-resistant patient samples. (**J**) Feature plot showing *CDH2* expression across different cell clusters.

Transcriptomic validation of the eight candidate hub genes was conducted using quantitative reverse-transcription PCR (qRT-PCR) in a paired ovarian cancer cell model comprising parental A2780 cells and their cisplatin-resistant counterpart, A2780/CP70. Among these genes, *CDH2* was significantly upregulated in A2780/CP70 cells compared to A2780 cells (*p* < 0.05), indicating a potential association with cisplatin resistance. Although *CAV1* and *SPP1* showed an upward expression in A2780/CP70 cells, these differences did not reach statistical significance (*p* > 0.05). In contrast, *FGF18* and *PDGFRA* were downregulated in A2780/CP70 cells relative to A2780 cells; however, none of these changes reached statistical significance (*p* > 0.05) (Figure 6A). Given its consistent and statistically significant upregulation, *CDH2* was selected for subsequent analyses and experimental validation.

The diagnostic value of *CDH2* expression in ovarian cancer was subsequently assessed. Receiver operating characteristic (ROC) curve analysis yielded an area under the curve (AUC) of 0.792 (*p* < 0.05), indicating a moderate diagnostic value (AUC = 0.8). Compared with established diagnostic and prognostic biomarkers for ovarian cancer CA125 (cancer antigen 125; mucin-16, MUC16), HE4 (human epididymis protein 4; WAP four-disulfide core domain protein 2, WFDC2), and PAX8 (paired box gene 8) as well as the broadly used surveillance biomarker CEA (carcinoembryonic antigen; CEACAM5), *CDH2* showed lower diagnostic performance than PAX8 (AUC = 0.983), CA125 (AUC = 0.993), and HE4 (AUC = 0.997) (all *p* <0.05), but outperformed CEA (AUC = 0.666, *p* < 0.05) (Figure 6B).

To further characterize changes in *CDH2* expression during chemotherapy in ovarian cancer, we cross-validated our findings using Single-cell RNA sequencing (scRNA-seq) data from clinical ovarian cancer samples (GSE211956). Based on chemotherapy response scores (CRS) from the clinical metadata, patients were classified into a resistant group (CRS1) and a sensitive group (CRS3). After batch correction, the two groups were well integrated in UMAP space, forming 12 distinct cell clusters (Figure 6C). Differential expression analysis of tumor cells, identified using the “FindMarkers” function, revealed that *CDH2* was significantly upregulated in the resistant group (expressed in 22.6% of cells), whereas its expression was low or undetectable in the sensitive group (*p* = 0.00087) (Figure 6D). Uniform Manifold Approximation and Projection (UMAP) feature plots and violin plots further confirmed the widespread distribution of *CDH2*-positive cells in the resistant group and their complete absence in the sensitive group (Figure 6E,F).

Subsequent analysis focusing on resistant patient samples (GSM6506108 and GSM6506109) identified 8 major cell clusters (Figure 6G), with *CDH2* expression detected across multiple cell types, particularly epithelial cells, smooth muscle cells, and endothelial cells, showing marked intracluster heterogeneity (Figure 6H). Targeted reanalysis of these three cell types yielded 5 distinct subclusters (clusters 0–4) (Figure 6I). *CDH2* expression was predominantly enriched in clusters 1 and 2, moderate in clusters 0 and 4, and absent in cluster 3 (Figure 6J). Marker gene analysis indicated that cluster 0 expressed *ABCG2*, cluster 1 expressed *KRT17* and *CXCL10*, and cluster 2 expressed *S100A1* (full marker lists in Supplementary Table S2A). These genes have been previously implicated in chemoresistance and tumor cell survival path- ways. Collectively, the single-cell transcriptomic analysis of clinical ovarian cancer samples suggests that elevated *CDH2* expression may contribute to cisplatin resistance by promoting epithelial–mesenchymal transition (EMT), enhancing cell–matrix interactions, and activating pro-survival signaling pathways.

### 2.6. Gene Set Enrichment Analysis (GSEA) Result and Validation

**Figure 7:**
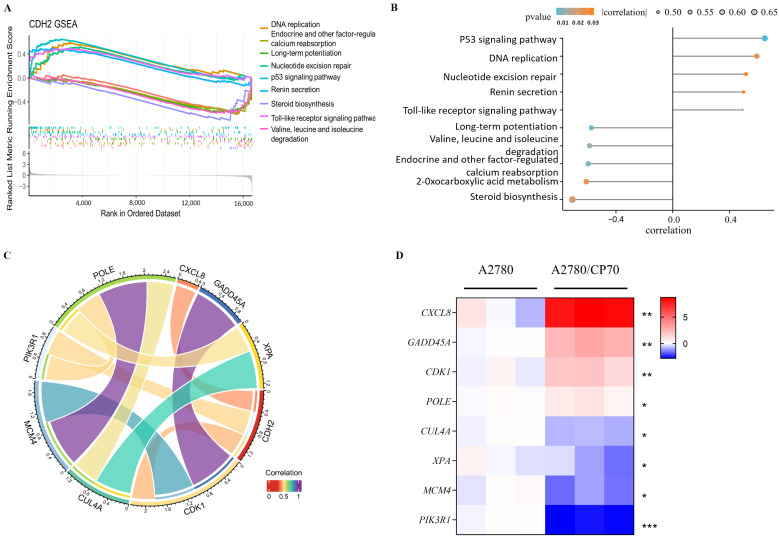
Single-gene GSEA and validation of *CDH2*-associated pathways in OC. (**A**) Bar plot showing the top five significantly enriched upregulated and downregulated pathways associated with *CDH2* expression, identified by single-gene GSEA. (**B**) Correlation analysis between *CDH2* expression levels and the enrichment scores of the identified pathways. (**C**) PPI chord diagram illustrating the relationships between *CDH2* and key genes within the upregulated pathways. (**D**) RT–qPCR validation of representative genes from the upregulated pathways in cisplatin-sensitive A2780 cells and cisplatin-resistant A2780/CP70 cells, with GAPDH used as the internal control. Data are presented as mean ± SD from three independent experiments; statistical significance was assessed using a paired *t*-test (* *p* < 0.05, ** *p* < 0.01, *** *p* < 0.001).

To comprehensively investigate the potential role of this hub gene, gene set enrichment analysis (GSEA) was performed using KEGG gene sets as the reference database. Single-gene GSEA indicated that *CDH2* upregulation was positively associated with the p53 signaling pathway, DNA replication, nucleotide excision repair, and Toll-like receptor (TLR) signaling pathway (Figure 7A), with detailed correlation coefficients shown in Figure 7B. Key genes within these pathways that are highly relevant to tumor drug resistance mechanisms (Supplementary Table S2B) were further analyzed. Chord diagram analysis revealed that *CDH2* interacted with critical effectors in the TLR signaling pathway (*CXCL8*, *PIK3R1*) and the p53 signaling pathway (*CDK1*) (Figure 7C).

Subsequent quantitative PCR (qPCR) validation in ovarian cancer cell lines demonstrated that the p53 pathway–related genes *CDK1* and *GADD45A* were significantly upregulated in A2780/CP70 cells compared with A2780 cells. Likewise, the TLR pathway gene *CXCL8* exhibited markedly higher expression in A2780/CP70 cells but minimal expression in A2780 cells (Figure 7D, probes detailed in Supplementary Table S2C). These findings suggest that *CDH2* may promote ovarian cancer progression and cisplatin resistance by modulating key biological processes, particularly through the p53 and TLR signaling pathways. Notably, the association between *CDH2* and the TLR pathway a central mediator of innate immune responses implies a potential role for *CDH2* in regulating tumor–immune interactions.

### 2.7. Connection of the Immune Cell Infiltration and Hub Genes CDH2

**Figure 8:**
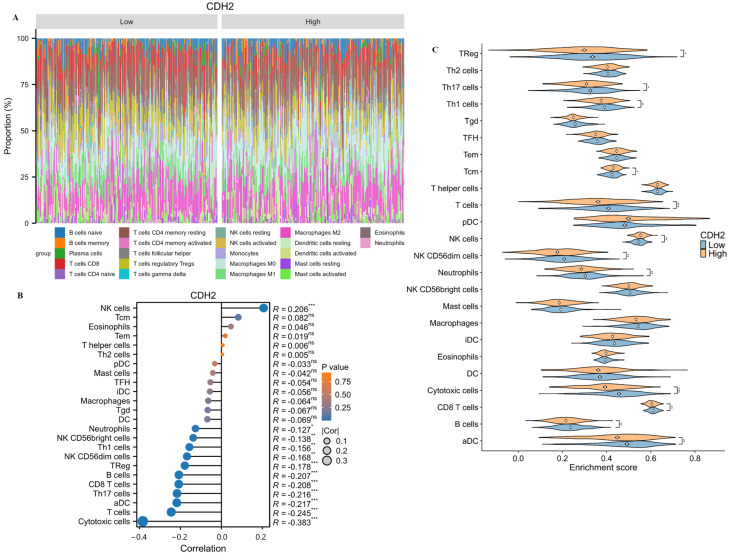
Immune cell infiltration patterns associated with *CDH2* expression in OC. (**A**) Stacked bar plot showing the relative proportions of 22 immune cell subtypes in each OC sample, estimated using the CIBERSORT deconvolution algorithm based on bulk transcriptomic data. (**B**) Correlation analysis between *CDH2* expression levels and the estimated infiltration of immune cell subsets, illustrating positive and negative associations across different immune populations. (**C**) Comparison of immune cell infiltration between high- and low-*CDH2* expression groups, demonstrating differences in the relative abundance of specific immune cell subsets. Statistical significance was determined as described in Section 4 (* *p* < 0.05, ** *p* < 0.01, *** *p* < 0.001 indicate significant differences).

To explore potential associations between immune cell infiltration and tumor-associated factors, the relative abundance of 22 immune cell subtypes in ovarian cancer samples was estimated using the CIBERSORT algorithm [25]. Comparison between the high- and low-*CDH2* expression groups revealed that tumors with higher *CDH2* expression exhibited a significantly higher proportion of activated natural killer (NK) cells within the tumor immune microenvironment (Figure 8A). Consistently, correlation analysis demonstrated a positive association between *CDH2* expression levels and activated NK cell infiltration.

In contrast, *CDH2* expression was negatively correlated with the infiltration of multiple immune cell populations, including cytotoxic cells, Th17 cells, activated dendritic cells, total T cells, CD8^+^ T cells, regulatory T cells (Tregs), B cells, and CD56^dim^ NK cells (Figure 8B). Further group comparisons showed that elevated *CDH2* expression was significantly associated with reduced infiltration of activated dendritic cells, B cells, cytotoxic cells, Tregs, Th17 cells, Th1 cells, total T cells, CD56^dim^ NK cells, and CD8^+^ T cells (Figure 8C), while the positive association with activated NK cells was retained. These findings suggest a potential link between *CDH2* expression and altered immune cell composition in ovarian cancer. However, given the limitations of computational deconvolution methods and the correlative nature of these analyses, these results should be interpreted cautiously and do not establish direct causal relationships.

### 2.8. Validation of CDH2 Expression in Clinical Specimens

**Figure 9:**
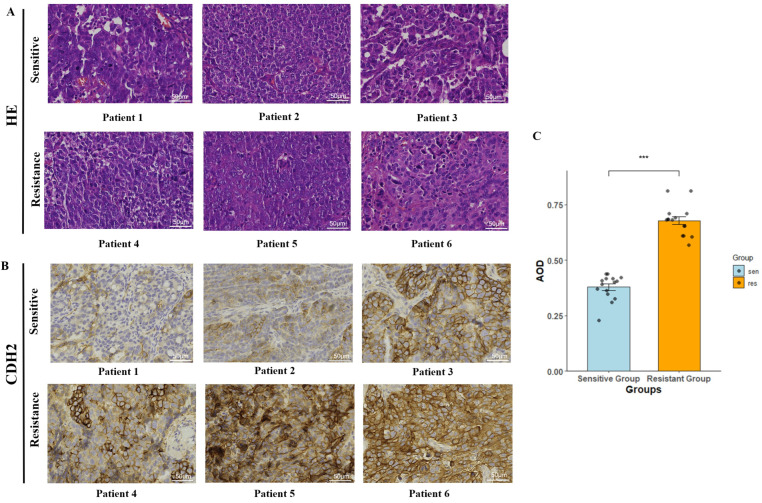
Validation of CDH2 expression in clinical OC specimens. (**A**) Hematoxylin and eosin (HE) staining of tumor sections from cisplatin-sensitive and cisplatin-resistant patients. Scale bar: 50 µm. (**B**) Immunohistochemical (IHC) staining showing CDH2 expression levels in tumor tissues from patients with differential cisplatin responses. Scale bar: 50 µm. (**C**) Quantification of CDH2 expression in first-surgery tumor tissues using AOD values. Statistical significance was assessed by unpaired *t*-test (*** *P* < 0.001 indicate significant differences).

To study the expression profile of CDH2 in OC tissues, protein levels were analyzed in clinical specimens from six cisplatin-sensitive and six cisplatin-resistant patients. Hematoxylin and eosin (HE)-stained sections were first examined under a light microscope to identify regions of tumor cell aggregation. Serial sections stained for CDH2 expression were used to image five randomly chosen high-power fields (400× magnification) inside the aggregation zones for each sample. According to qualitative analysis, tissues from patients who were resistant to cisplatin showed higher and more widespread CDH2 immunostaining, while samples from patients who were sensitive to cisplatin showed lower expression levels (Figure 9B). Image-Pro Plus software (version 6.0.0.260) was used to quantitatively analyze the images, measuring the stained area and integrated optical density (IOD). CDH2 expression levels were represented by the average optical density (AOD), which is computed as IOD divided by the area. According to the data, the cisplatin-resistant group’s CDH2 protein expression was noticeably greater than that of the cisplatin-sensitive group (Figure 9C) (the AOD value are detailed in Supplementary Table S1H).

### 2.9. Association Between CDH2 Expression and Drug Sensitivity

**Figure 10:**
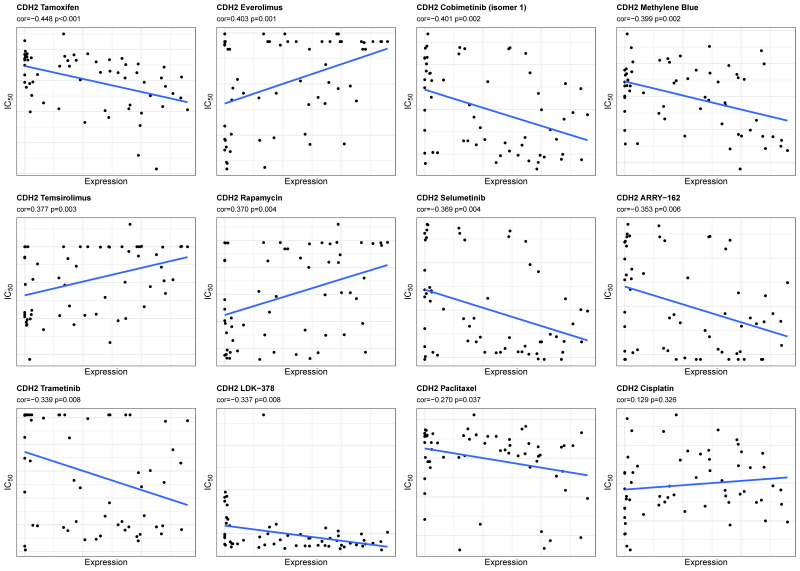
Correlation between *CDH2* expression and drug sensitivity in the NCI-60 cell line panel. Pearson correlation analysis was performed between *CDH2* mRNA expression levels and log_10_ (IC_50_) values for ten compounds. Compounds with correlation coefficient |r| > 0.30 and *p* < 0.05 were considered highly associated with *CDH2*-related drug sensitivity. Blue lines represent linear regression trends. Tamoxifen, Selumetinib, Trametinib, and Cobimetinib exhibited the strongest negative correlations with *CDH2* expression, whereas Paclitaxel and Cisplatin showed no significant associations. These analyses are exploratory, based on the NCI-60 panel with limited ovarian cancer representation, and should be interpreted as hypothesis-generating.

**Figure 11:**
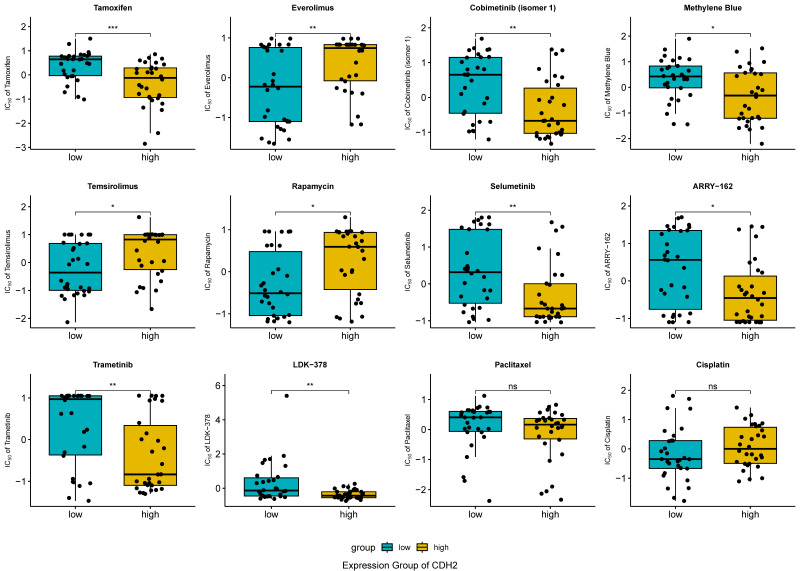
Comparison of drug sensitivity between *CDH2*-high and *CDH2*-low expression groups. Box plots depict log_10_ (IC_50_) values for compounds stratified by median *CDH2* expression. Compounds with correlation coefficient |r| > 0.30 and *P* < 0.05 in Pearson correlation analysis were considered highly associated with *CDH2*-related drug sensitivity. These analyses are exploratory, based on the NCI-60 panel with limited ovarian cancer representation, and should be interpreted as hypothesis-generating (* *p* < 0.05, ** *p* < 0.01, *** *p* < 0.001 indicate significant differences).

To investigate potential association between *CDH2* expression and drug sensitivity, pharmacogenomic analysis was performed using the CellMiner database. Pearson correlation and group-based comparisons identified ten compounds with nominally significant associations associated with *CDH2* expression (*p* < 0.05) (Figure 10). Among these, Tamoxifen, Selumetinib, Trametinib, and Cobimetinib (isomer 1) exhibited lower half-maximal inhibitory concentration (IC_50_) values in the *CDH2* high-expression group compared with the low-expression group (Supplementary Table S3, Figure 11). Selumetinib (r = −0.369, *p* = 0.004) and Trametinib (r = −0.339, *p* = 0.008) showed significant negative correlations with *CDH2* expression, with similar trends for the other two compounds. No significant associations were observed between *CDH2* expression and paclitaxel (r = −0.270, *p* = 0.037; IC_50_ not significantly different) or cisplatin (r = 0.129, *p* = 0.326), standard first-line chemotherapeutics for ovarian cancer. These results are exploratory and intended to generate hypotheses rather than indicate direct therapeutic applicability.

### 2.10. Molecular Docking Verification of CDH2 with Related Drug Small Molecule Components

**Figure 12:**
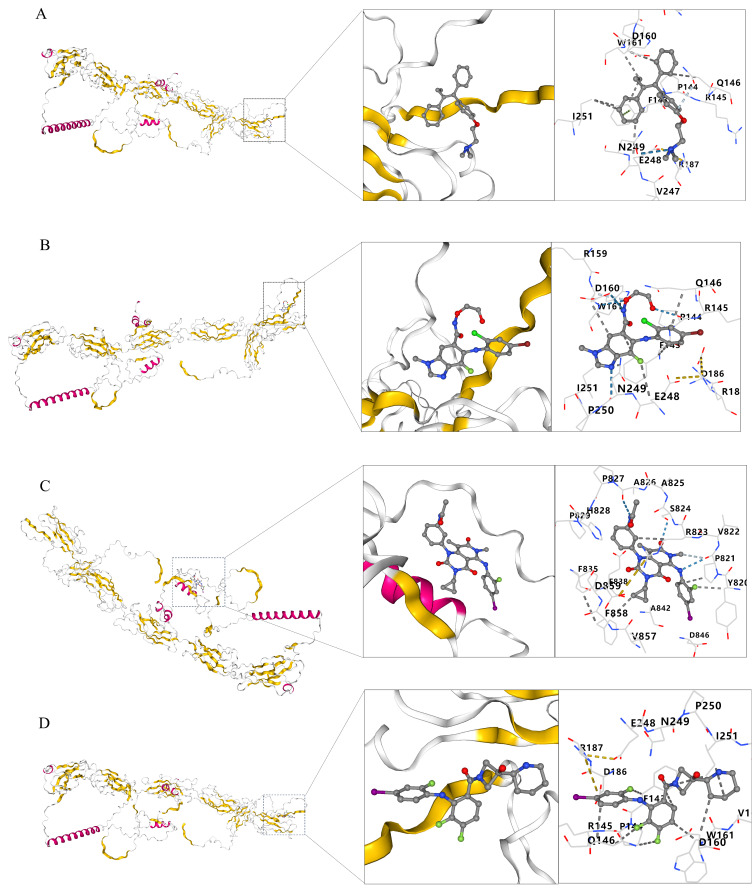
Molecular docking of candidate compounds with CDH2. Schematic diagrams depict the predicted binding of each compound to the AlphaFold-modeled CDH2 structure. Enlarged panels highlight the binding pockets, with colored dashed lines indicating potential weak interactions between each ligand and the corresponding amino acid residues. (**A**) Tamoxifen, (**B**) Selumetinib, (**C**) Trametinib, (**D**) Cobimetinib. These docking analyses are exploratory and hypothesis-generating, and do not provide direct evidence of druggability or clinical applicability.

Molecular docking analyses were performed to assess potential binding interactions between CDH2 and the four candidate compounds using CB-DOCK2. Tamoxifen exhibited a docking score of −6.7 within a 1282 Å^3^ cavity, with 48% of pocket residues showing high or very high pLDDT confidence (Figure 12A). Selumetinib (score: −7.7; cavity: 1282 Å^3^) had 50% high-confidence residues, though 40% of residues showed low confidence, indicating pocket flexibility (Figure 12B). Trametinib showed the strongest predicted affinity (score: −8.4) within a smaller, less confident pocket (596 Å^3^), dominated by low-to-medium pLDDT residues (Figure 12C). Cobimetinib (score: −8.4; cavity: 1282 Å^3^) bound to a pocket containing 45% high-confidence residues, with 40% low-confidence residues (Figure 12D). These results indicate that all four compounds show favorable theoretical binding affinities toward CDH2, with several pockets supported by high-confidence structural regions. These results suggest favorable theoretical interactions in silico and provide hypothesis-generating insights for potential CDH2-related compound interactions, without implying direct druggability or clinical applicability.

## 3. Discussion

Cisplatin-based chemotherapy remains the cornerstone of OC treatment; however, the development of cisplatin resistance continues to severely compromise therapeutic efficacy and patient prognosis. Although EMT has long been implicated in the acquisition of chemoresistance [26,27,28,29], most prior studies have treated EMT as a broad phenotypic state rather than interrogating the contribution of individual EMT-associated genes to specific resistance mechanisms. In this study, we employed an integrated bioinformatic and experimental strategy to identify *CDH2* (N-cadherin) as a hub gene associated with cisplatin resistance and prognosis in OC.

### 3.1. CDH2 as a Clinically Relevant Marker of Platinum Resistance and Prognosis

CDH2 (N-cadherin), a calcium-dependent transmembrane glycoprotein, is fundamentally involved in mediating cell–cell adhesion and tissue morphogenesis [30]. Beyond its established role as a canonical marker of EMT [31], aberrant *CDH2* expression has been implicated in the aggressive progression of various malignancies, including lung, liver, and gastric cancers, primarily by enhancing cellular migratory and invasive capacities [32,33,34,35]. In OC, accumulating evidence supports the prognostic significance of *CDH2*. For instance, large-scale tissue microarray analyses have identified high cytoplasmic N-cadherin expression as a significant predictor of increased recurrence risk and diminished disease-free survival (DFS) [36]. Complementing these clinical observations, our study demonstrates that *CDH2* expression is notably elevated in cisplatin-resistant OC cell lines and clinical specimens. Beyond its general association with mesenchymal phenotypes, our data highlight *CDH2* as a clinically pertinent node within the resistance landscape, supported by its moderate diagnostic performance (AUC = 0.792). We interpret these findings with necessary caution, recognizing that an AUC of this magnitude indicates a promising yet moderate discriminatory capacity. Thus, while *CDH2* represents a potential biomarker for identifying platinum-refractory states, its clinical utility warrants further refinement and validation in larger, independent multicenter cohorts to transition from an exploratory finding to a robust diagnostic tool.

### 3.2. Refining EMT Paradigms: From Phenotypic Switch to Resistant Subpopulations

Unlike prior EMT-focused studies that conceptualize cisplatin resistance as a uniform mesenchymal state, our single-cell RNA sequencing analysis reveals that *CDH2* expression is selectively enriched in specific resistant tumor subpopulations rather than uniformly across all malignant cells. This observation aligns with recent evidence indicating that cisplatin-resistant OC cells adopt distinct survival strategies, including altered spheroid formation and peritoneal invasion, mediated in part by *CDH2*-dependent adhesion dynamics [37]. These findings suggest that *CDH2* may contribute to the formation of a “survival niche”, supporting clonal persistence under chemotherapeutic stress rather than merely reflecting global EMT activation.

### 3.3. CDH2 and On-Target Resistance: A Hypothesis-Generating Link to DNA Repair and Stress-Response Pathways

A key contribution of this work is the identification of a previously underappreciated association between *CDH2* expression and on-target cisplatin resistance pathways, particularly nucleotide excision repair (NER) and DNA replication. While EMT is typically linked to off-target survival signaling (e.g., PI3K/AKT or anti-apoptotic pathways), our GSEA and qPCR validation indicate that *CDH2*-high tumors are enriched for transcriptional programs related to DNA damage repair. This observation is consistent with experimental models of acquired cisplatin resistance demonstrating coordinated upregulation of *CDH2* alongside DNA repair proteins such as XRCC1 and PARP1 [38].

Following cisplatin-induced DNA damage, the p53 pathway acts as a pivotal arbiter of apoptosis and genomic stability. Its dysregulation, often coupled with impaired nucleotide excision repair mechanisms, constitutes a hallmark of chemoresistance in ovarian cancer [39,40]. Concurrently, aberrant TLR signaling has been implicated in fostering an immune-evasive and pro-survival microenvironment [41]. Notably, our findings that *CDH2* expression correlates with both p53 and TLR pathways suggest that it is integrated within a broader stress-adaptive transcriptional landscape. While these associations remain correlative, we cautiously hypothesize that *CDH2* may function as a regulatory scaffold or upstream modulatory node. Potentially through its cytoplasmic domain or associated protein complexes, *CDH2* may fine-tune the transcriptional responsiveness of DNA damage and immune-related stress pathways, rather than serving as a direct initiator. This conceptual framework is consistent with contemporary perspectives that reclassify EMT-associated molecules, including *CDH2*, as gatekeepers of cellular plasticity that intersect with DNA repair competence rather than acting as canonical damage sensors [42]. Furthermore, recent evidence identifying ANXA4 as a modulator of *CDH2*-associated programs to attenuate cisplatin-induced apoptosis [43] provides additional mechanistic plausibility for this model. Collectively, these observations suggest that *CDH2* represents a critical molecular intersection between cell-surface adhesion and intracellular resistance programs, though its precise regulatory hierarchy warrants further experimental dissection.

### 3.4. Immune Infiltration Patterns Associated with CDH2 Expression

The tumor immune microenvironment is increasingly recognized as a determinant of chemotherapy response [44,45]. Our immune infiltration analyses, based on CIBERSORT deconvolution of bulk transcriptomic data, revealed that higher *CDH2* expression was associated with altered immune cell composition, including increased activated NK cells and reduced infiltration of cytotoxic T cells, regulatory T cells, B cells, and dendritic cells. These findings suggest a potential association between *CDH2* expression and immune contexture remodeling. In addition, no significant correlation was observed between *CDH2* and the abundance of M1 or M2 macrophage subtypes, suggesting a potentially selective role of *CDH2* in modulating specific immune cell populations. Given the inherent limitations of computational deconvolution in capturing the full spectrum of macrophage plasticity, these immune-related findings are presented as exploratory. Thus, while *CDH2* expression is associated with altered immune landscapes, the present data do not support a definitive role for *CDH2* as an immune-modulatory driver.

### 3.5. Exploratory Drug Sensitivity and Molecular Docking Analyses

Our pharmacogenomic analyses using the CellMiner database identified correlations between *CDH2* expression and sensitivity to several targeted agents. It is important to note that CellMiner analyses are based on the NCI-60 panel, which includes limited representation of ovarian cancer cell lines. Consequently, these findings should be considered exploratory and hypothesis-generating rather than predictive of clinical efficacy. Similarly, molecular docking analyses suggesting potential interactions between *CDH2* and selected compounds are purely in silico and do not imply direct druggability or therapeutic applicability without functional validation. These analyses nonetheless provide a conceptual bridge for future mechanistic and translational investigations.

### 3.6. Limitations and Future Directions

Several limitations of this study warrant consideration. First, although supported by qRT-PCR, immunohistochemistry, and independent scRNA-seq validation, much of the analysis relied on publicly available datasets with limited sample size and clinical heterogeneity. Second, qRT-PCR validation was restricted to a single cisplatin-sensitive/resistant cell line pair, limiting generalizability. Third, due to clinical constraints, matched primary and recurrent tumor tissues were unavailable; therefore, IHC validation was performed on a small cohort of primary surgical specimens (n = 6), constraining statistical power. Fourth, immune infiltration and drug sensitivity analyses were computational and correlative, and molecular docking was exploratory. Finally, *CDH2* was validated only at the expression level, and its functional role in cisplatin resistance requires further mechanistic investigation through in vitro and in vivo studies.

## 4. Materials and Methods

### 4.1. Data Information and Preprocessing

We obtained mRNA expression data and treatment information, GSE73935 [23], from the GEO database (https://www.ncbi.nlm.nih.gov/geo/ (accessed on 28 July 2023)) [46], which is built on GPL13667 platform. After filtering the GEO datasets with the keywords ‘’ovarian cancer cisplatin resistance”, a series consisting of 48 samples from GSM1906460 to GSM1906508 was selected. Subsequently, the data were subjected to a screening process based on their clinical characteristics, resulting in the identification of 27 samples from GSM1906460- GSM1906486 as cisplatin resistant (CR, n = 6), doxorubicin resistant (DO, n = 6), paclitaxel resistant (PX, n = 6), topotecan resistant (TO, n = 6), and control group (CON, n = 3). The samples were classified into five distinct categories. The data set was then merged, normalized and filtered using the limma package of the R language, resulting in the generation of an expression matrix.

### 4.2. Analysis of Differential Gene Expression

Gene expression differences between cisplatin-treated and control samples were analyzed using the limma package in R. Differentially expressed genes (DEGs) were identified based on an absolute log_2_ fold change ≥ 1 and an adjusted *p*-value < 0.05. Multiple testing correction was consistently applied using the Benjamini–Hochberg false discovery rate (FDR) method across all DEG analyses.

### 4.3. Weighed Gene Co-Relation Network Analysis

WGCNA was performed on the normalized expression matrix from the GSE73935 dataset [23] by BIC-bioinfo platform (https://www.bic.ac.cn/BIC/ (accessed on 31 July 2023)). The main expression data and sample attribute matrix, including treatment information, were pasted into the text area and then submitted to the program. The necessary parameters were set, and the program was subsequently run. Because each sample in GSE73935 was treated with a single chemotherapeutic agent, module–trait correlations were analyzed independently for each drug-specific resistance phenotype.

#### 4.3.1. Construction of the Co-Expression Network

The analytical pipeline commenced with clustering of initial gene expression profiles using WGCNA, during which transcriptional outlier detection and exclusion were performed to ensure data integrity. The fit index and the average connectivity of the scale-free network are then calculated, and an appropriate soft threshold β = 24 is selected. Simultaneously, the neighbor-joining matrix and TOM between genes are calculated simultaneously, and a hierarchical clustering tree between genes is established according to the TOM. Applying dynamic tree-cutting heuristics, the transcriptional network was partitioned into 27 distinct co-expression modules, with chromatic coding assigned to each functional unit.

#### 4.3.2. Correlation Between Modules and Phenotypes Analysis

Module-phenotype correlation heatmaps facilitate the detection of co-expressed gene clusters strongly associated with clinical phenotypes, along with quantitative evaluation of their statistical significance. It can be observed that the green-yellow and blue blocks exhibit a strong positive correlation with cisplatin resistance, while the pink block displays a negative correlation. The *p*-value for these correlations is less than 0.05, indicating a statistically significant association. The color blocks designated as dark orange and purple exhibit a strong positive correlation with paclitaxel resistance, while the color block designated as light cyan demonstrates a strong negative correlation. Each sample in the GSE73935 dataset was exposed to a single chemotherapeutic agent only, and no samples received combined treatments. Therefore, module–trait correlations were assessed independently for each drug-specific resistance phenotype without confounding effects from other chemotherapeutic agents.

#### 4.3.3. Identifying Hub Genes

Transcriptional candidates derived from strongly correlated co-expression modules were overlapped with preidentified DEGs, generating a composite gene cohort for subsequent interactome interrogation. This integrated gene set underwent systematic protein association mapping through the STRING platform (https://string-db.org/ (accessed on 18 November 2023)), with resultant interaction networks subjected to topological analysis. Network visualization and computational characterization were implemented in Cytoscape, where node centrality quantification was executed via the CytoHubba extension using the MCC scoring metric. Molecular candidates ranking within the octet of highest MCC values were designated as putative molecular regulators, hypothesized to encode pivotal proteins governing network architecture dynamics.

### 4.4. GO and KEGG Pathway Enrichment Analysis

To delve deeper into the main biological effects of the selected hub genes, we employed the Metascape online platform (https://metascape.org/gp/index.html (accessed on 22 November 2023)) to conduct GO enrichment analysis on these genes. The gene list should be uploaded and the species designated as H. sapiens. The custom analysis should then be undertaken, with the KEGG pathway selected under pathway in enrichment, after which the program may be executed. The analytical outputs should thus be downloaded and plotted as long bar graphs via the ggplot toolkit in R statistical computing environment. The list of genes to be analyzed was then subjected to a GO enrichment analysis using the colorspace, stringi, data.table, ggplot2, tidyverse, ggvenn, DOSE, clusterProfiler, enrichplot, and org.Hs.eg.db packages in R. The resulting *p*-values, with a q-value set to 0.05, were visualized using the ggplot2 package to generate the first ten results.

### 4.5. Hub Genes Expression Level and Kaplan–Meier Survival Analysis

Transcriptomic validation of candidate molecular regulators was conducted using the GEPIA plat- form (http://gepia.cancer-pku.cn/ (accessed on 23 July 2024)), with differential expression patterns cross-validated between OC specimens and their histologically normal counterparts. Analytical stringency was maintained using threshold parameters defined as absolute |logFC| > 1 combined with Benjamini–Hochberg *p*-value less than 0.05. PFS assessments were conducted using the KM-Plotter platform (version 2023, https://kmplot.com/analysis/ (accessed on 23 July 2024)), which aggregates transcriptomic data from 1436 OC patients across 14 GEO datasets (GSE14764, GSE15622, etc.) and 1 TCGA dataset. Patients were stratified by expression level of hub-genes using the platform’s auto-select optimal cutoff. Survival differences were evaluated using log- rank analysis, with hazard ratios (HR) estimated via Cox regression. Statistical significance was defined as a two-sided Benjamini–Hochberg adjusted *p* < 0.05. Subgroup analyzes were performed according to histological grade. To minimize confounding effects from key clinical variables, survival analyses were conducted within a relatively homogeneous patient subgroup. Specifically, only patients with high-grade serous ovarian cancer at stage IV who had received platinum-based chemotherapy were included. Patients with other histological subtypes, earlier stages, or missing treatment information were excluded. Given the restricted and homogeneous cohort, additional multivariate adjustment for clinical covariates such as stage and grade was not performed.

### 4.6. Cell Lines and Culture

Two ovarian adenocarcinoma models were employed: the parental A2780 strain (Cell Resource Center, IBMS/CAMS&PUMC, Beijing, China) and its cisplatin-resistant derivative A2780/CP70 (generously provided by Dr. Alwyn Dart, Cardiff China Medical Research Collaborative, Cardiff University, Cardiff, UK). Cellular propagation was conducted under standard culture conditions using F12/DMEM nutrient mixture (Gibco, 12400024) containing 10% heat-inactivated FBS (Vistech, VSE-1000-500. Distributor: Beijing Nano-Ace Technology Co., Ltd., Beijing, China) and 1% antibiotic cocktail (Corning, 30-002-CI. Distributor: Beijing Yuhengfeng Technology Co., Ltd., Beijing, China). A 5% CO_2_-humidified environment at 37 °C was used to culture the cells, with medium replenishment every 48 h.

### 4.7. Quantitative Reverse-Transcription PCR (qRT-PCR) Analyses

Total RNA was extracted from all cell lines by the AM1561 kit (Invitrogen™ Distributor: Beijing Jiuyu Jintai Biotechnology Co., Ltd., Beijing, China) following the manufacturer’s protocol. RNA integrity was assessed via NanoDrop by measuring OD_260/280_ and OD_260/230_ ratios.

Quantitative PCR was performed on Applied Biosystems 7500 Fast Real-Time PCR System in 15 µL reactions containing 1 µL cDNA, 6.5 µL nuclease-free water, 5 µL primer mix, and 6.5 µL PowerUp™ SYBR™ Green Master Mix (A25742, Applied Biosystems, Thermo Fisher Scientific (China) Co., Ltd., Shanghai, China). The thermal cycling protocol was as follows. 50 °C for 120 s. Then 10 min at 95 °C. Followed by 40 amplification cycles. Each cycle included 95 °C for 15 sec and 60 °C for 60 sec. Data were normalized and visualized using GraphPad Prism 8. The primers were synthesized by Sangon Biotech (Beijing, China). Primer sequences detail is listed in Supplementary Table S1G.

### 4.8. Diagnostic ROC Curve Analysis

Transcript-level RNA-seq data, uniformly processed by the Toil pipeline and expressed in TPM format, were downloaded from the UCSC Xena platform (https://xenabrowser.net/datapages/ (accessed on 10 July 2025)) for both the TCGA and GTEx projects. Ovarian serous cystadenocarcinoma samples were extracted from the TCGA cohort, while normal ovarian tissue samples were obtained from the GTEx dataset. The clinical diagnostic value of CDH2 was assessed by constructing ROC curves using the pROC package in R, and results were visualized with the ggplot2 package.

### 4.9. Clinical Sample–Derived Single-Cell RNA-Seq Dataset

scRNA-seq dataset GSE211956 was obtained from the GEO database. The expression matrix and associated metadata were imported into Seurat to construct a Seurat object. As quality control had already been performed by the data provider, the Seurat object was split by sample, and the functions NormalizeData, FindVariableFeatures, ScaleData, and RunPCA were applied sequentially for normalization, identification of highly variable genes, data scaling, and principal component analysis (PCA). Tumor cells were defined based on canonical epithelial markers and SingleR-based annotation, thereby distinguishing tumor cells from stromal and other non-malignant cell populations, followed by manual verification. Batch effects were corrected using the IntegrateLayers function with the “harmony” method, and the integrated low-dimensional embeddings were used for downstream visualization. As the data were obtained from publicly available databases, no additional approval from the institutional ethics committee was required.

### 4.10. Gene Set Enrichment Analysis (GSEA) of Single Gene

Single-gene GSEA uses correlation analysis between the target gene and the dataset genes to obtain a dataset with a high correlation with the target gene, and then uses this dataset for enrichment analysis to predict the possible functions of its target gene, which can be conveniently further verified by experiments. Single gene GSEA were performed in R environment by packages such as ggplot2, limma, ggsci, clusterProfiler, enrichplot, patchwork, org.Hs.eg.db, selecting a *p* value of 0.05, maxGSSize = 200, minGSSize = 10, pvalueCutoff = 0.05 and nPerm = 10,000. Finally, the first five pathways were visualized.

### 4.11. Immune Infiltration Analysis

Immune cell infiltration profiling was performed on the TCGA-OV, ovarian cancer cohort retrieved from TCGA repository. Single-sample gene set enrichment analysis (ssGSEA) was implemented through the Xiantao Academic online platform (https://www.xiantao.love/ (accessed on 20 August 2024)) to quantify immune cell components, with normal tissue specimens systematically excluded from the analytical pipeline.

### 4.12. Immunohistochemistry (IHC)

Surgically resected ovarian cancer (OC) tissues were obtained from the pathology archive of Yijishan Hospital, Wannan Medical College (WNMC), and grouped according to clinical characteristics. This study was approved by the Ethics Committee of Yijishan Hospital, affiliated with WNMC, Wuhu, China (Approval No. 202234), and all procedures were conducted in accordance with institutional ethical guidelines. OC tumors that recurred within six months after primary surgery and cisplatin-based chemotherapy were defined as cisplatin-resistant, as previously described [47]. No recurrence was observed within the same time frame in the cisplatin-sensitive group.

Paraffin-embedded tumor tissues from six cisplatin-resistant and six cisplatin-sensitive OC patients were subjected to immunohistochemical staining to evaluate CDH2 protein expression. Tissue specimens were fixed in 10% neutral-buffered formalin, routinely dehydrated, paraffin-embedded, and sectioned at a thickness of 4 μm. After deparaffinization and rehydration, antigen retrieval was performed, followed by incubation with 3% hydrogen peroxide to block endogenous peroxidase activity. Sections were then blocked with normal serum and incubated with a primary antibody against N-cadherin (CDH2; Abcam, Trumpington, UK, ab76011) at a dilution of 1:100 for 2 h at room temperature (28 °C). Subsequently, sections were incubated with the corresponding secondary antibody and visualized using diaminobenzidine (DAB) as the chromogen. Hematoxylin was used for nuclear counterstaining, and slides were mounted with neutral resin. CDH2 expression was quantified using an automated image analysis approach based on AOD, minimizing potential bias associated with manual scoring. Random high-power fields were selected in a standardized manner for all samples. Image acquisition and analysis were performed in a blinded manner with respect to clinical information.

### 4.13. Drug Sensitivity Analysis

Drug sensitivity analysis was performed using the CellMiner database (https://discover.nci.nih.gov/cellminer/home.do (accessed on 25 November 2025)), which contains activity profiles for 889 compounds across the NCI-60 cancer cell line panel, including 563 agents in clinical trials and 326 FDA-approved drugs. Pearson correlation analysis was applied to assess the associations between gene expression levels and the log_10_IC_50_ values of each drug. Compounds showing significant correlations (correlation coefficient > 0.30 and *p* < 0.05) were considered highly associated with gene-related drug sensitivity. The relationships between selected drugs and gene expression were subsequently visualized using boxplots and correlation plots. As ovarian cancer is underrepresented within this panel, the observed correlations between gene expression and drug sensitivity should be interpreted cautiously.

### 4.14. Molecular Docking

Molecular docking analysis was conducted to evaluate the interactions between candidate compounds and the target protein. The predicted three-dimensional structure of the protein was retrieved from the AlphaFold Protein Structure Database (https://alphafold.ebi.ac.uk/ (accessed on 25 November 2025)). Docking simulations were performed using the CB-DOCK2 platform (https://cadd.labshare.cn/cb-dock2/ (accessed on 28 November 2025)) with default parameters. Importantly, the docking results are considered exploratory and hypothesis-generating, and do not provide direct evidence of druggability or clinical applicability.

### 4.15. Data and Statistical Analysis

All statistical computations were executed in GraphPad Prism v9.5.1 (GraphPad Software, La Jolla, CA, USA) and RStudio (v2022.07.1+554; R Foundation for Statistical Computing). Experimental data were expressed as mean SEM. Intergroup comparisons of normally distributed continuous variables employed two-tailed unpaired Student’s *t*-tests. DEG profiles and AOD quantifications were processed through R-based analytical pipelines, with a threshold of *p* < 0.05 was used to determine significance for all bidirectional hypothesis testing.

## 5. Conclusions

In conclusion, this study identifies *CDH2* as a clinically significant hub gene that converges with cisplatin resistance and poor prognosis in ovarian cancer. By integrating bulk, single-cell, and preliminary experimental analyses, we demonstrate that *CDH2* marks a distinctive resistant state characterized by enhanced DNA repair capacity, stress-response signaling, and phenotypic plasticity. Although our findings remain exploratory and do not establish a direct causal axis, they identify a critical intersection between membrane-associated adhesion, genomic integrity, and therapeutic evasion. This work provides a foundation for future studies to evaluate whether *CDH2* serves as a functional biomarker or a targetable therapeutic vulnerability in refractory ovarian cancer.

## Data Availability

The data used for bioinformatics analysis in this study can be accessed in the NCBI Gene Expression Omnibus (GEO http://www.ncbi.nlm.nih.gov/geo/ (accessed on 28 July 2023)) under accession numbers GSE73935, GSE211956. The materials and codes used and/or analyzed during the current study are available from the corresponding author on reasonable request.

## Supplementary Materials

The following supporting information can be downloaded at: https://www.mdpi.com/article/10.3390/ijms27020713/s1.
